# MRTF/SRF dependent transcriptional regulation of TAZ in breast cancer cells

**DOI:** 10.18632/oncotarget.7333

**Published:** 2016-02-11

**Authors:** Chen-Ying Liu, Siew Wee Chan, Fusheng Guo, Aleksandra Toloczko, Long Cui, Wanjin Hong

**Affiliations:** ^1^ Institute of Molecular and Cell Biology, Agency for Science, Technology and Research (A*STAR), Proteos, Singapore 138673, Singapore; ^2^ Department of Colorectal and Anal Surgery, Xinhua Hospital, Shanghai Jiaotong University School of Medicine, Shanghai 200092, China

**Keywords:** Hippo pathway, TAZ, MRTF/SRF, Heregulin β1 (HRG1), breast cancer

## Abstract

Dysregulation of Hippo pathway results in activation of transcriptional co-activators YAP/TAZ in breast cancer. Previously, we showed that overexpression of TAZ in breast cancer promotes cell migration, invasion and tumorigenesis. Here, we show that upregulation of TAZ in breast cancers could also be due to dysregulation of TAZ transcription. Heregulin β1 (HRG1) increases TAZ mRNA level in breast cancer cells. TAZ is a direct target of MRTF/SRF transcriptional factors which are activated by HRG1. Both MRTF/SRF and TAZ are the important downstream effectors enhancing cell migration induced by HRG1. TAZ mRNA level is correlated with nuclear localization of MRTF in breast cancer cells and the mRNA level of MRTF/SRF direct target genes in breast cancers, indicating the correlation between MRTF/SRF activity and TAZ expression. Our results provide new insights into the transcriptional regulation of TAZ and dysregulation mechanism of TAZ in breast cancer, which could be a new therapeutic strategy for breast cancer.

## INTRODUCTION

The Hippo pathway is a conserved signaling pathway regulating the organ size in mammalians [1]. Dysregulation of the Hippo pathway occurs in multiple cancers, including breast cancer [1]. Main components of Hippo pathway consist of the core kinases MST1/2 and LATS1/2 with their binding partners WW45 and MOB1, respectively. Activation of the Hippo pathway results in cytoplasmic retention and degradation of downstream transcriptional co-activator YAP/TAZ, leading to the repression of TEAD-mediated transcription and inhibition of cell proliferation. Multiple extracellular stimuli have been found to regulate the Hippo pathway, including contact inhibition [2], mechanotransduction [3] and GPCR [4]. The human ERBB receptor family members are tyrosine kinase receptors which play important roles in the progression of various cancers [5]. Heregulins are a group of EGF-like ligands for surface ERBB2, ERBB3 and ERBB4 which are often overexpressed in the breast cancers [5] and promote cell proliferation [6] and cell migration [7], in association with tumorigenesis and aggressive phenotypes [8].

Recently, Heregulin β1 (HRG1) pathway has been reported to be correlated with the Hippo pathway in that the intracellular cytoplasmic domain fragment (ERBB4-ICD) can interact with YAP/TEAD and enhance YAP target gene expression [9]. Here, we show that the expression of TAZ, another Hippo pathway effector, can be induced by the HRG1 which mediates the biological function of HRG1 in breast cancer cells. HRG1 promotes TAZ mRNA expression through activating the MRTF/SRF pathway. TAZ is a direct target gene of MRTF/SRF in breast cancer in which the mRNA expression level of TAZ is correlated with the MRTF nuclear localization and the classic direct target genes of MRTF/SRF. Our results provide a new mechanism of TAZ transcriptional regulation and therapeutic strategy for the treatment of breast cancer.

## RESULTS

### TAZ is the downstream effector enhancing cell migration induced by Heregulin β1 in breast cancer cells

**Figure 1:**
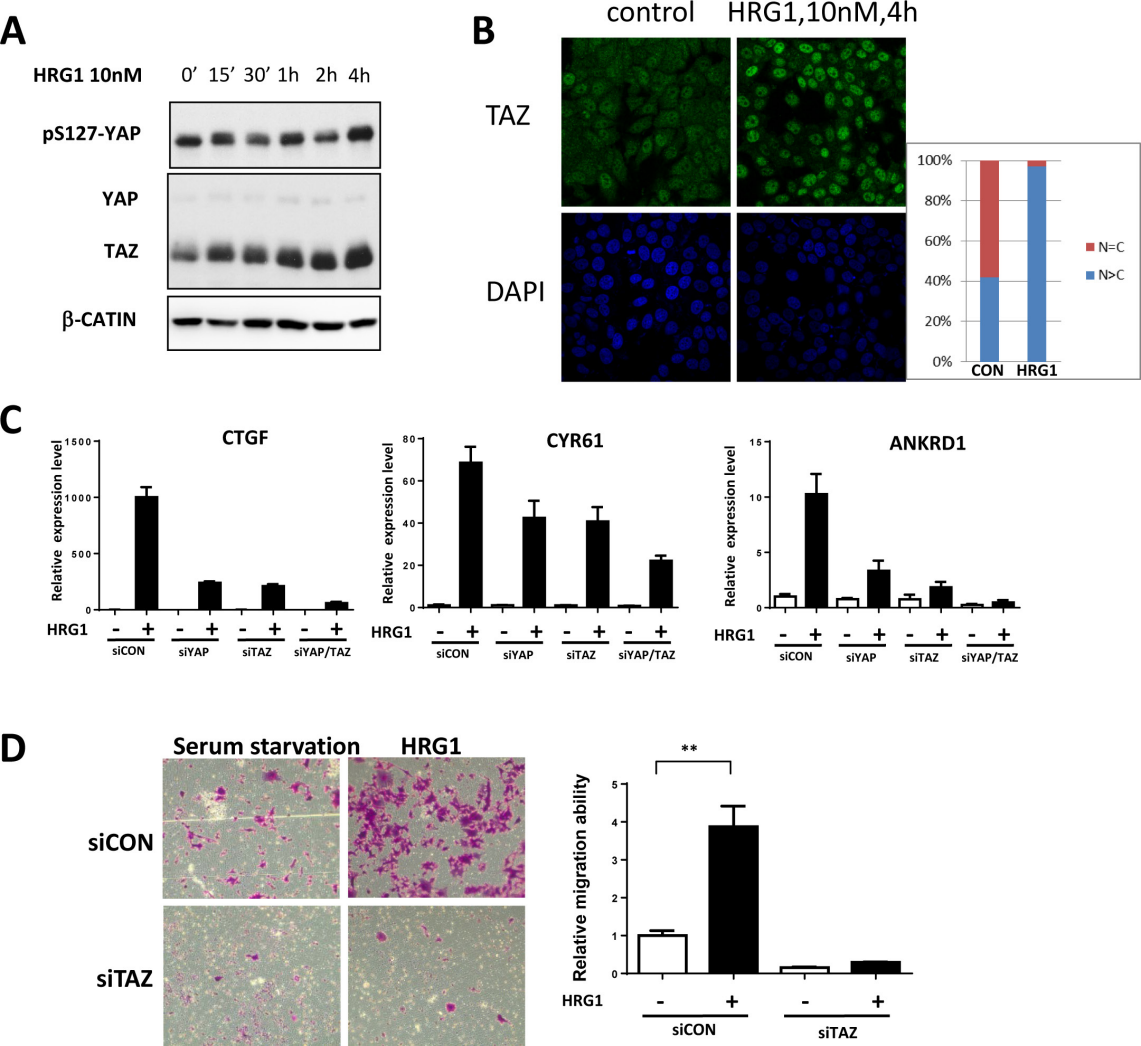
TAZ is activated by HRG1 in breast cancer cells (**A**) HRG1 induced increasing TAZ protein level in MCF7 cells. MCF7 cells were serum starved for overnight, then treated with 10 nM HRG1 with the indicated time. Cell lysates were processed for western blotting using antibodies against the indicated proteins. (**B**) HRG1 increased TAZ protein level and promoted TAZ nuclear localization. Immunofluorescent staining of TAZ in the serum starved and HRG1 treated MCF7 cells were performed. (**C**) Knockdown of TAZ reduced the expression of Hippo target genes induced by HRG1 in MCF7. Cells were transfected with indicated siRNA for two days and were changed to serum free medium for overnight. On the third day, samples were collected after 1 h HRG1 treatment. (**D**) Knockdown of TAZ blocked cell migration induced by HRG1. Cells were transfected with TAZ siRNA for two days and serum-starved for overnight. The cell migration was analyzed by transwell with or without HRG1 (10 nM) for 48 h. **P < 0.01 by Student's *t*-test.

We also noticed the upregulation of CTGF and CYR61 genes by HRG1 from the public microarray data [10] and therefore explored the underlying mechanisms. Consistent with previous report, HRG1 treatment in MCF7 cells didn't affect YAP protein level and only slightly increase the phosphorylation level of YAP [9]. However, interestingly, TAZ protein level was significantly increased after 4 hours HRG1 treatment (Figure 1A). Consistent with a previous report [11], partially nuclear localization of TAZ was observed in the serum starved MCF7 cells (Figure 1B). Immunostaining of TAZ showed increasing intensity and nuclear localization after HRG1 treatment (Figure 1B), indicating the activation of TAZ by HRG1. Next, we asked whether activation of TAZ really mediating the function of HRG1 and activating downstream gene expression. Either knockdown of YAP or TAZ dramatically reduced the gene expression of CTGF, CYR61 and ANKRD1 induced by HRG1, while simultaneous knockdown of both YAP and TAZ almost totally abolished the target gene activation (Figure 1C). Furthermore, knockdown of TAZ not only reduced the basal level of migration ability of MCF7 cells but also blocked cell migration enhanced by HRG1 (Figure 1D). These results suggest that TAZ is induced and activated by HRG1 to enhance tumorigenesis in breast cancer cells.

### MRTF/SRF promotes TAZ mRNA expression in breast cancer cells

**Figure 2:**
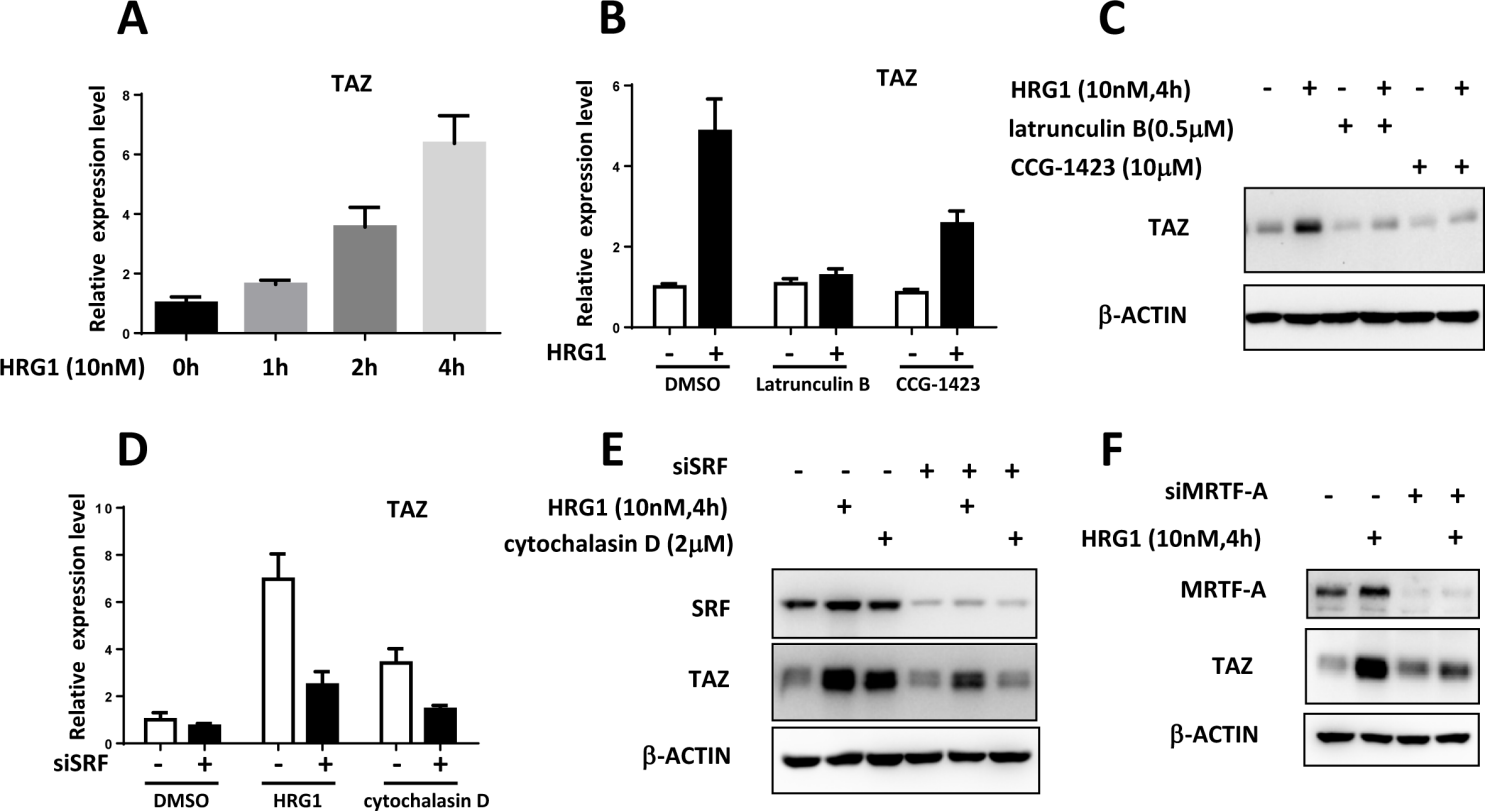
Transcriptional regulation of TAZ by MRTF/SRF in breast cancer cells (**A**) HRG1 promoted TAZ transcription in MCF7 cells. qPCR analysis of TAZ mRNA level in the MCF7 cells after HRG1 treatment for the indicated time. (**B**) Inhibition of RhoA dependent transcription blocked the TAZ induction by HRG1 in MCF7 cells. MCF7 cells were serum starved for overnight. Before HRG1 treatment, the cells were pre-incubated with Latrunculin B and CCG-1423 for 1 h. TAZ mRNA levels were measured. (**C**) Western blot analysis of TAZ protein level in MCF7 cells treated with indicated compounds. (**D**) Knockdown of SRF suppressed induction of TAZ mRNA by HRG1 and Cytochalasin D in MCF7 cells. Cells were transfected with SRF siRNA for two days, then serum starved for overnight. HRG1 treatment was performed on the third day. (**E**) Western blot analysis of the TAZ protein level in the MCF7 cells with SRF knockdown and HRG1 treatment. (**F**) Knockdown of MRTF-A inhibited the TAZ induction by HRG1 in MCF7. Experiments were performed like SRF knockdown as described above.

Next, we explored the mechanism of TAZ induction by HRG1 in breast cancer cells. Analysis of half-life of TAZ protein showed HRG1 didn't increase the protein stability in MCF7 cells (Figure S1A). Intriguingly, TAZ mRNA level was increased up to 6 folds after HRG1 treatment, indicating a transcriptional regulation of TAZ by HRG1 (Figure 2A). Pre-incubation of pan-ERBB inhibitor lapatinib suppressed TAZ induction by HRG1 at both mRNA and protein levels (Figure S1B, S1C). Interestingly, EGFR inhibitor AG-1478 also blocked the upregulation of TAZ mRNA level, but EGF treatment couldn't induce TAZ expression (Figure S1D), implicating a heterodimer of ERBB mediating the function of HRG1 in activating TAZ expression in breast cancer cells. Recently, HIF1α has been found to enhance TAZ transcription [12] and ERBB4 can bind HIF1α to promote HIF1α dependent transcription [13]. Thus, we tested whether HRG1 induced ERBB4-ICD can increase TAZ expression level through activation of HIF1α. However, both a HIF1α inhibitor and a γ-Secretase inhibitor which can inhibit the production of ERBB4-ICD induced by HRG1 failed to block TAZ induction by HRG1 (Figure S1B, S1C).

RhoA activation by HRG1 has been reported before [14] which is also involved in inhibiting the Hippo pathway through promoting the actin polymerization [1]. Myocardin-related transcription factor (MRTF)-mediated transcriptional activation through the serum response factor (SRF) is activated in response to actin polymerization by RhoA [15]. We found that actin polymerization inhibitor (Latrunculin B) and a newly developed RhoA-dependent transcription inhibitor (CCG- 1423) can suppress the induction of TAZ by HRG1 at mRNA and protein level (Figure 2B, 2C). Interestingly, cytochalasin D, the MRTF/SRF activator, could upregulate TAZ expression in MCF7 cells. Knockdown of SRF in MCF7 abolished the TAZ induction by both HRG1 and cytochalasin D (Figure 2D, 2E). Knockdown of MRTF-A was also sufficient for inhibiting TAZ upregulation by HRG1 in MCF7 (Figure 2F). These results suggest that MRTF/SRF transcription factors activated by actin polymerization are required for the TAZ transcriptional activation by HRG1 in breast cancer cells.

### MRTF/SRF pathway is activated by HRG1 in breast cancer cells

**Figure 3:**
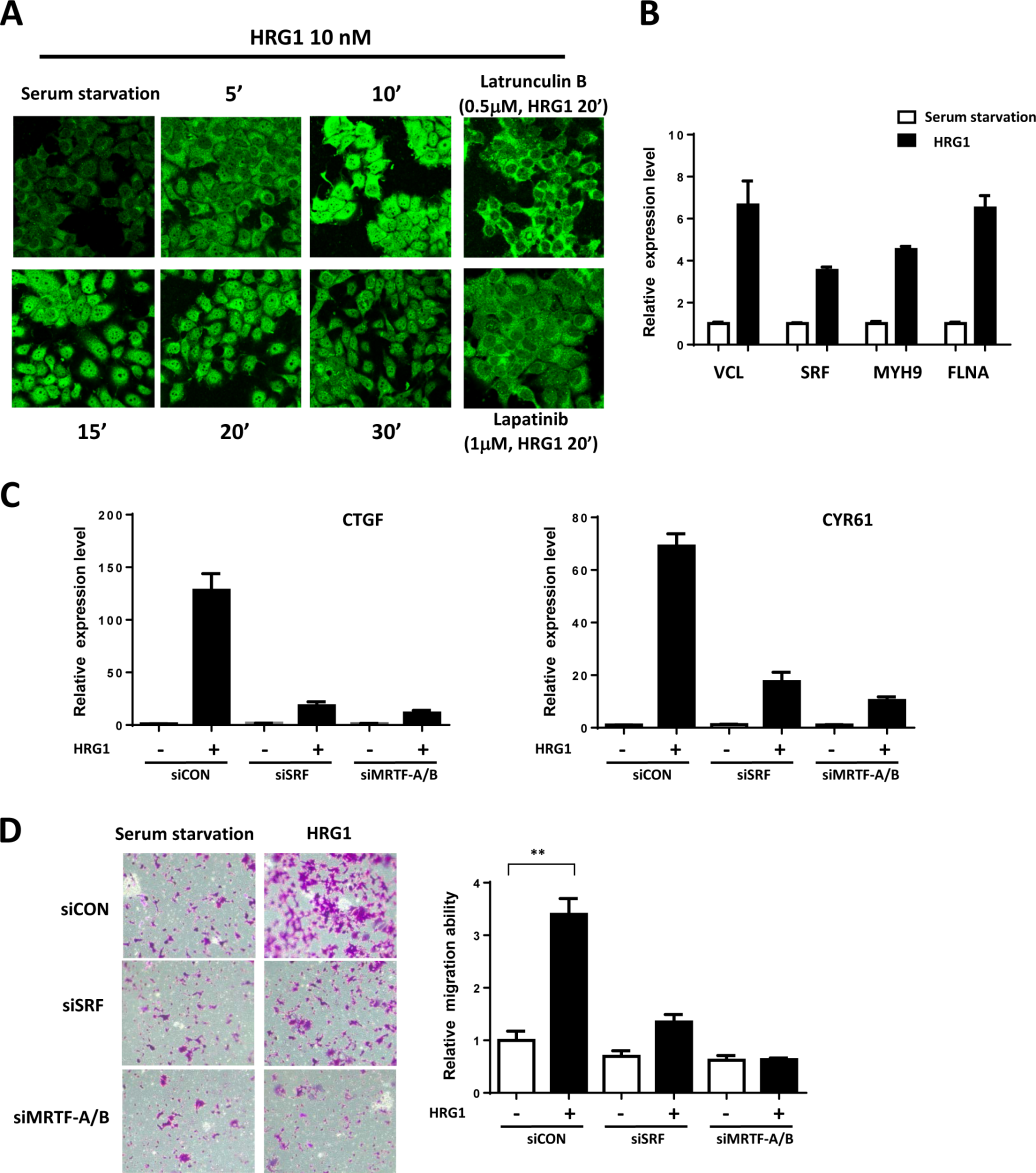
MRTF/SRF are activated by HRG1 in breast cancer cells (**A**) HRG1 promoted MRTF-A nuclear translocation through ERBB activation and promoting actin polymerization. MCF7 cells were serum-starved and pre-treated with indicated compounds, and then treated with HRG1 for indicated time. Cells were fixed and stained with MRFT-A antibody. (**B**) HRG1 activated the expression of MRTF/SRF target genes. Serum starved MCF7 cells were treated with HRG1 for 4 h. qPCR was performed to detect the expression of indicated genes. (**C**) Knockdown of SRF and MRTF-A/B suppressed the Hippo target genes' induction by HRG1. Cells were transfected with SRF or MRTF-A/B siRNA for two days and serum-starved for overnight. Samples were collected after 1 h HRG1 treatment. (**D**) Knockdown of SRF or MRTF-A/B blocked cell migration induced by HRG1. Cells were transfected with SRF or MRTF-A/B siRNA for two days and serum-starved for overnight. The cell migration was analyzed by transwell with or without HRG1 (10 nM) for 48 h. **P < 0.01 by Student's *t*-test.

To show the activation of MRTF/SRF pathway, we stained the MRTF-A in the MCF7 cells treated with HRG1. In serum starved cells, MRTF-A mainly located in the cytoplasm (Figure 3A). HRG1 treatment induced a rapid nuclear localization of MRTF-A in 10 minutes and strong nuclear staining of MRTF-A was observed in 20 minutes (Figure 3A). Treatment of lapatinib and Latrunculin B blocked the nuclear translocation of MRTF-A (Figure 3A), indicating the involvement of ERBB receptor activation and actin polymerization induced by HRG1. Actin microfilament effectors are the downstream target genes of MRTF/SRF [15]. VCL, FLNA, MYH9 and SRF, which are direct target genes, were all upregulated by HRG1 at mRNA level (Figure 3B), further indicating the activation of MRTF/SRF dependent transcription. Knockdown of SRF and MRTF inhibited the induction of CTGF and CYR61 (Figure 3C). Also, knockdown of SRF and MRTF blocked the cell migration induced by HRG1 (Figure 3D). These results suggest that MRTF/SRF is activated by HRG1 and mediates the biological function of HRG1 in activating TAZ in breast cancer cells.

### TAZ is a direct target of MRTF/SRF in breast cancer cells

**Figure 4:**
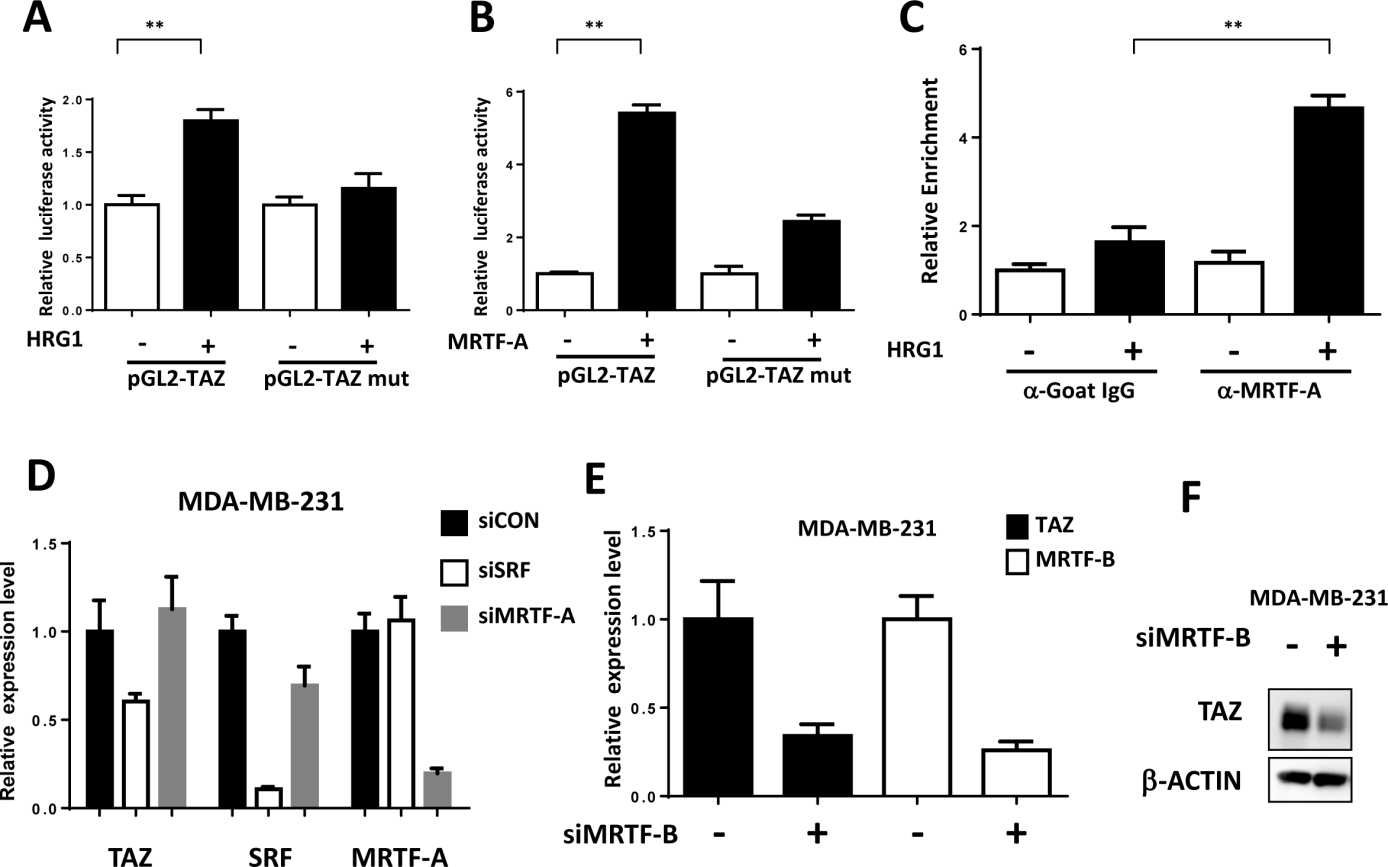
TAZ is a direct target of MRTF/SRF in breast cancer cells (**A**) HRG1 increased luciferase activity of TAZ promoter reporter. Luciferase activity of TAZ WT and CArG box mutant reporter were co-transfected with SV40-Rellina into the MCF7 cells. After 6 h transfection, medium was changed to serum free medium for overnight. 24 h later, cells were treated with HRG1 for 4 h and the relative luciferase activity was detected. **P < 0.01 by Student's *t*-test. (**B**) MRTF-A enhanced the luciferase activity of TAZ promoter reporter. TAZ reporters were co-transfected with MRTF-A expression plasmids as indicated in 293 cells. 24 h later, the relative luciferase activities were detected. **P < 0.01 by Student's *t*-test. (**C**) HRG1 promoted MRTF-A binding to the TAZ promoter. Serum starved and HRG1 treated (30 min) MCF7 cells were collected for ChIP-qPCR assay. ChIP assay were performed as previously described [28]. qPCR primers were designed based on the promoter sequence around the CArG box. **P < 0.01 by Student's *t*-test. (**D**) Knockdown SRF decreased the TAZ expression in MDA-MB-231. qPCR was performed to detect TAZ mRNA level in the cells transfected with indicated siRNA for 3 days. (**E, F**) MRTF-B promotes TAZ transcription in MDA-MB-231 cells. MDA-MB-231 cells were transfected with MRTF-B siRNA for 72 h. qPCR and western blot analysis were performed to detect the TAZ and MRTF-B level.

Next, we explored whether MRTF/SRF can directly activate TAZ transcription. Three mRNA isoforms of TAZ with two different transcriptional starting sites have been found in human genome although all encoding the same protein. ChIP-seq data of the active transcriptional epigenetic markers and POLA2 in the MCF7 from Cistrome database [16] all indicated the isoforms with shorter 5′UTR were the TAZ transcripts expressed in the MCF7 cells (Figure S2A). Interestingly, ChIP-seq of SRF also showed the direct binding of SRF at the same transcriptional starting site (Figure S2A), further sequence analysis revealed a SRF consensus motif, the CArG box, in the TAZ promoter (Figure S2B). We generated the TAZ luciferase reporter with WT promoter and CArG box mutant one. HRG1 increased the relative TAZ luciferase activity but not the CArG mutant one in MCF7 cells (Figure 4A). Moreover, overexpression of MRTF-A was sufficient to enhance the TAZ luciferase activity which was abolished by CArG box mutation (Figure 4B). ChIP-PCR analysis further showed that, in serum starved cells, MRTF-A didn't bind to the TAZ promoter (Figure 4C); however, HRG1 induced a rapid binding of MRTF-A to the TAZ promoter, likely resulting in the TAZ expression through activating SRF (Figure 4C).

### Correlation between TAZ and MRTF/SRF pathway in breast cancers

**Figure 5:**
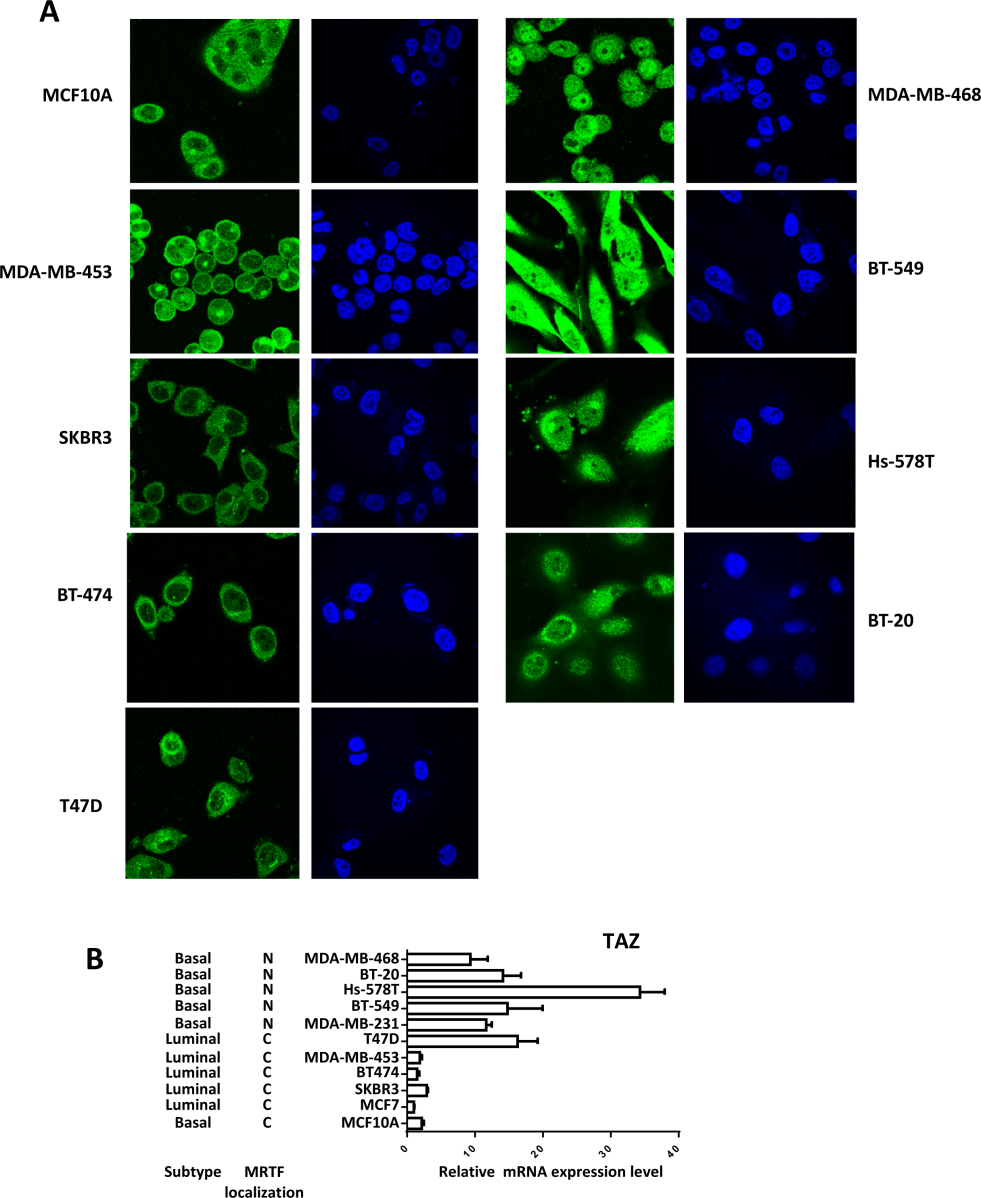
High TAZ expression level is correlated with nuclear enrichment of MRTF in breast cancer cell panel (**A**) Multiple breast cancer cells were cultured in the normal culture medium with 10% FBS according to the ATCC guidelines, then fixed and stained with MRTF-A. (**B**) The TAZ expression levels in the breast cancer cells were detected by qPCR. Relative expression levels were normalized to the TAZ expression level in MCF7 cells.

**Figure 6:**
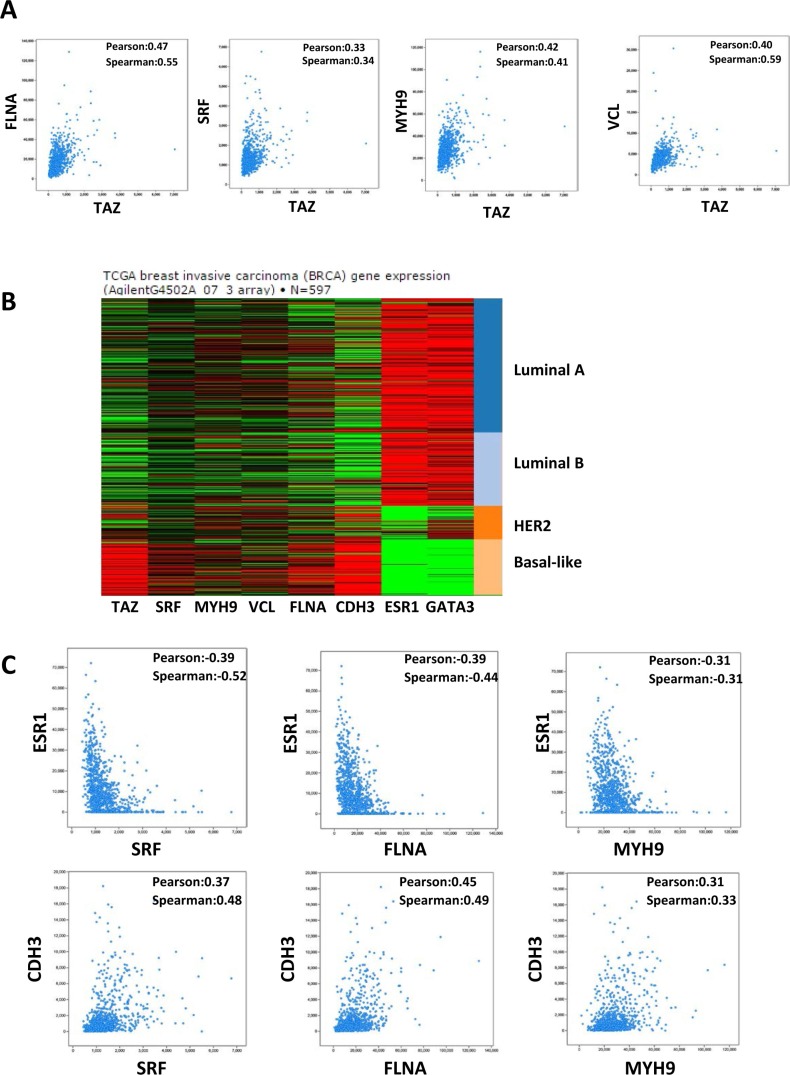
Correlation between TAZ and MRTF/SRF pathway in the TCGA breast cancer database (**A**) TAZ expression level is correlated with the expression level of MRTF/SRF target genes in breast cancer. Co-expression data between TAZ and FLNA, SRF, MYH9, and VCL, respectively, in the breast cancer TCGA RNA-seq data were extracted from the cbioportal database. The value of Pearson's correlation coefficient and Spearman's correlation coefficient were generated by the cbioportal database to measure the correlation of gene co-expression. (**B**) The expression levels of TAZ and MRTF/SRF target genes in the TCGA datasets from UCSC Cancer genomics browser were shown. (**C**) The expression levels of MRTF/SRF target genes are positively associated with basal marker CDH3 and negatively correlated with luminal marker ESR1. Co-expression data in the breast cancer TCGA RNA-seq data were extracted from the cbioportal database.

MRTF/SRF pathway was first delineated in the fibroblast and then found to be involved in the cancer metastasis including breast cancer [17]. Previous study found that MRTF is predominantly localized in the nucleus of metastatic MDA-MB-231 breast cancer cells [17]. To explore whether MRTF/SRF can activate TAZ in other breast cancer cells, knockdown of SRF and MRTF were performed in MDA-MB-231 cells. SRF knockdown decreased the TAZ expression level (Figure 4D). Knockdown of MRTF-B but not MRTF-A significantly decreased the TAZ expression at mRNA and protein level (Figure 4E, 4F), indicating MRTF-B was the predominant MRTF promoting TAZ expression in MDA-MB-231 cells. Next, we stained the MRTF subcellular localization in multiple breast cancer cell lines and compared to mRNA level of TAZ (Figure 5A, 5B). In all cell lines with TAZ low expression, MRTF-A was mainly present in the cytoplasm. In the cell lines with TAZ high expression (MDA-MB-231, MDA-MB-468, BT-549), strong nuclear localization of MRTF was observed. Two cell lines (Hs-578T, BT- 20) with weak nuclear staining and one (T47D) with cytoplasmic staining of MRTF-A were observed. Gene expression data from TCGA extracted from the cbioportal database [18] also showed significant correlation between TAZ expression level and the level of MRTF/SRF target genes (Figure 6A). High expression level of TAZ mRNA was associated with basal-like breast cancers [19] (Figure 5B), similarly, the expression level of MRTF/SRF target genes were higher in the basal-like breast cancers compared to other subtypes of breast cancers (Figure 6B). Furthermore, MRTF/SRF target genes' expression was positively associated with basal marker (CDH3), but negatively correlated with luminal marker (ESR1) (Figure 6C), suggesting that the activity of MRTF/SRF is higher in basal-like breast cancers than other subtypes. All these data suggest a transcriptional regulation of TAZ by MRTF/SRF in breast cancers.

## DISCUSSION

Multiple downstream effectors have been found to be involved in mediating the function of heregulin activation, including enhancing YAP activity [5, 9]. Our findings show a new mechanism that heregulin pathway can cross-talk with the Hippo pathway at the transcriptional level by upregulating TAZ. Interestingly, heregulin β1 was found to induce GPR30/GPER upregulation in breast cancer cell [20], which can function as a GPCR activating the YAP/TAZ [21], and TAZ can promote ERBB ligands expression including HRG1 [22]. Thus, heregulin can activate YAP/TAZ activity through multiple ways in breast cancer, probably forming an autocrine positive feedback loop. Besides, in this study, we also show that MRTF/SRF is activated by heregulin. Both Hippo pathway and MRTF/SRF pathway are the downstream effectors of heregulin activation in breast cancers.

Post-transcriptional regulation of Hippo pathway has been widely studied; however transcriptional regulation of Hippo pathway components is still not well explored. For YAP and TAZ, most of the post-transcriptional regulation mechanisms are similar. Given the different phenotypes of YAP and TAZ knockout mice [23, 24], the *in vivo* biological function of YAP/TAZ and the spatial-temporal control of YAP/TAZ transcription could be different. Different transcriptional mechanisms could also render ways to differentially regulate YAP and TAZ *in vivo*. Hypoxia can promote TAZ expression through activating HIF1α [12]. Here, we show that heregulin enhances TAZ transcription by activating MRTF/SRF. Thus, like activation of the Hippo pathway by multiple extracellular stimuli, different stimuli can also regulate TAZ expression through different transcription factors.

TAZ protein expression is a prognostic marker for multiple cancers, including breast cancer [25, 26]. However, TAZ mRNA expression is associated with poor prognosis in basal-like breast cancers [19] which indicates that, besides post-modification regulation by Hippo pathway, dysregulation of TAZ mRNA expression also results in high expression of TAZ in breast cancers. Previous studies suggest that high expression level of TAZ in breast cancer probably results from copy number amplification [19, 27]. Here, we found high expression of TAZ in breast cancer was correlated with high mRNA level of MRTF/SRF target genes indicating the dysregulation of TAZ in breast cancer could also be due to the dysregulation of TAZ transcription by MRTF/SRF. Thus, targeting the transcription of TAZ could be a potential therapeutic strategy for breast cancer.

## MATERIALS AND METHODS

### Cell lines and compounds

Breast cancer cell lines MCF7, T47D, BT-474, SKBR3, MCF10A, MDA-MB-453, MDA-MB-231, MDA-MB-468, BT-549, Hs578T, BT-20 were purchased from ATCC and cultured as ATCC guidelines. All compounds used in this study were purchased from Selleck.

### Transfection

siRNA transfection were performed by using lipofectamine RNAi MAX reagent as the manufacturer's guide. The following siRNA were used for gene knockdown: YAP, L-012200-00-0005; TAZ, L-016083-00-0005; SRF, L-009800-00-0005, MRTF-A, L-015434-00-0005; MRTF-B, GTAACAGTGGGAATTCAGC.

### Western blot

Cells were lysed in the NP-40 cell lysis buffer (50 mM Tris-HCl, 150 mM NaCl, 1% NP-40, 50 mM NaF, 1 mM Na_3_VO_4_, 1 mM PMSF with protease inhibitor cocktail). Antibodies YAP/TAZ (CST: 8418), pS127-YAP (CST: 4911), SRF (CST: 5417), MRTF-A (Santa Cruz: sc-21558) and β-ACTIN (Santa Cruz: sc-47778 HRP) were used for western blot.

### Immunofluorescent staining

Experiments were performed as previously described [28]. Briefly, cells were fixed by 4% PFA for 1 h and permeabilized with 0.1% Triton X-100 for 10 min. After blocking with 3% BSA in PBS for 30 min, cells were incubated with the first antibody for 1 h at RT, following incubation with the FITC-conjugated second antibody. DAPI was used for nuclear indication. TAZ (BD: 560235) and MRTF-A (Santa Cruz: sc-21558) were used to stain the TAZ and MRTF-A.

### qPCR

RNA was extracted by using the RNeasy Mini Kit. cDNA was rever-transcribed by using the PrimeScript RT Master Mix. qPCR was performed using the SYBR green reagents.

### qPCR primers used in this study

WWTR1 (F: GGCTGGGAGATGACCTTCAC, R: C TGAGTGGGGTGGTTCTGCT);

CTGF (F: AGGAGTGGGTGTGTGACGA, R: CC AGGCAGTTGGCTCTAATC);

CYR61 (F: AGCCTCGCATCCTATACAACC, R: TT CTTTCACAAGGCGGCACTC);

ANKRD1 (F: CACTTCTAGCCCACCCTGTGA, R: CCACAGGTTCCGTAATGATTT);

SRF (F: AGAGGTGCTAGGTGCTGTTTGGAT, R: TGAGTGCCACTGGCTTTGAAGAGA);

MRTF-A (F: CTCCAGGCCAAGCAGCTG, R: CC TTCAGGCTGGACTCCAC);

MRTF-B (F: CTTCCTGTGGACTCCAGTG, R: TG TGACTCCTGACTCGCAG);

VCL (F: TCAGATGAGGTGACTCGGTTGG, R: G GGTGCTTATGGTTGGGATTCG);

MYH9 (F: CTAAGAGCCTCGCCAAGC, R: GT CTTCTCCAGCTCCTGTC);

FLNA (F: TGTCACAGGTGCTGGCATCG, R: CG TCACTTTGCCTTTGCCTG);

### Chromatin immunoprecipitation

Experiments were performed as previously described [28]. MRTF-A antibody (Santa Cruz: sc-21558) and control goat IgG were used for immunoprecipitation. The ChIP-enriched DNA was subjected to qPCR using promoter-specific primers: TAZ ChIP (F: TCTCCAGTG ACAGAGGCACTT, R: ACAAGGCCAGCTTTTCCAC).

### Luciferase assay

MRTF-A expression plasmid was purchased from Addgene (11978). TAZ promoter was amplified by PCR and inserted into pGL2-basic vector. CArG box mutant was generated by using the QuikChange Site-Directed Mutagenesis Kit.

The primers used for TAZ promoter amplification and CArG box mutant:

TAZ promoter (F: GGGGTACCCGCACCCTCT CTACTTCCAG, R: GAAGATCTAGTCTAAGGGCTTCG GCTCT);

CArG box mutant (F: CAAGATGCCTCCTCGCC AGATTAAATATAATCACAAGAGCTAAGCAG, R: CTG CTTAGCTCTTGTGATTATATTTAATCTGGCGAGGAG GCATCTTG).

### Cell migration assay

MCF7 cells were serum starved for overnight, the next day 5*10^4^ cells were added into the upper chamber (CORNING transwell, 8 μm pore) in serum free medium with or without HRG1 (10 nM). Culture medium with 10% FBS were added into the lower chamber to create the chemotaxis. Cell migration ability was analyzed after 48 h.

## SUPPLEMENTARY MATERIAL FIGURES


