# Supplementary material for: MRTF/SRF dependent transcriptional regulation of TAZ in breast cancer cells
Source: Oncotarget. 2016 Feb 11;7(12):13706–16. doi: 10.18632/oncotarget.7333 (PMC4924672; doi:10.18632/oncotarget.7333)
Supplement: Supplementary file 1 [file oncotarget-07-13706-s001.pdf]

MRTF/SRF dependent transcriptional regulation of TAZ in breast cancer cells

Supplementary Materials

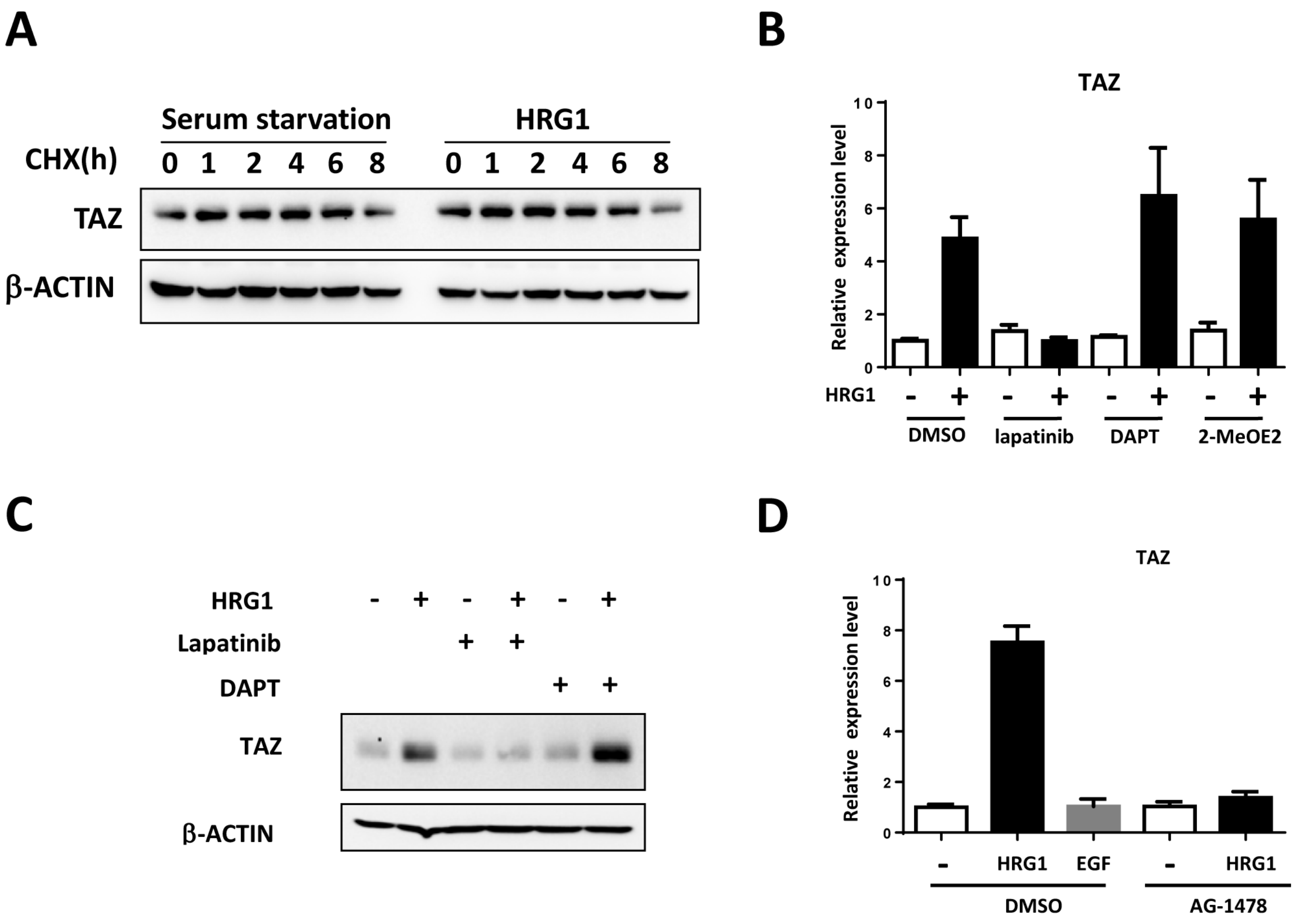

Supplementary Figure S1: Protein stability control, ERBB4-ICD and HIF1α are not involved in the TAZ induction by HRG1 in breast cancer cells. (**A**) HRG1 didn't affect TAZ stability. MCF7 cells were serum-starved for overnight. After 1 h HRG1 treatment, cells were treated with CHX for indicated time. (**B, C, D**) Activation of ERBB receptor, but not the EGF, production of ERBB4-ICD and HIF1α were required for the TAZ induction by HRG1. Cells were treated or pre-treated with indicated compounds for 1 h, then with HRG1 for 4 h. lapatinib (1 μM), DAPT (10 μM), 2-MeOE2 (100 μM), AG-1478 (1 μM), EGF (50 ng).

A

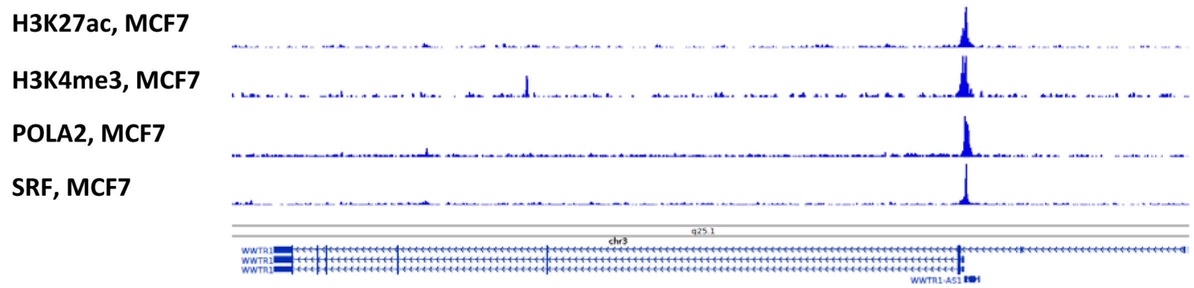

B

SRF consensus motif: CC (A/T)$_6$ GG

TAZ promoter : CCAAATATGG

MUT promoter: TTAAATATAA

```
GGCACTTTCTGGGCACCGTCTGTGCTCTAGCAGACAATTGAGAGCCAGGC    -601
AGCCAAGATGCCTCCTCGCCAGACCAAATATGGTCACAAGAGCTAAGCAG    -551
CCTCTCGTGGAAAAGCTGGCCTTGTTGGTGTCTCCCCGCGCCGGGGCGCA    -501
CCCGGGCCGGCCTTCGGGCAGAATCGCGGGTCAGTGCGGCGCTCCGGGTG    -451
CTGCAGGCCTCGGCGCCTGGCTGCAGGGGAGGGCGCGGTGTCCAGGACCG    -401
GCCCTTCCTAGGCCTGAGCCCCTCATCCGCGTGCCCTCCGCCACCCCCGG    -351
CCTTGGTTTCGGTCCACAACCCCTTGATGAAACAGGCTCAGGCCACTTTC    -301
CCTTTGATCCCGCCCCAAGTCCGTGGTAAACTCAAAGGAATGCAGATGCA    -251
GGTCTGAGCGGGAGAGGTCCGCGCGGCCGGGAGGAGTGGGTGGGGGGCAC    -201
CCGTCTCCTCTTACCCAGTAAAGTACCCATCACGCCCAGGGTTTTCTGGA    -151
GCCGAGGTGGGAGGAGGAGGAGGAGGAAGAGGAGGGGCGAGCGGGGGCTG    -101
GCCGGCTAGGGACTAGGAGGTCATACATAATTCAACAGCTCAACTTTCGG    -51
GCCCGCCTCTTTCCTGGGGGTGGGAGTTTGCTCCAAACTTTGTTTATGGG    -1
```

Supplementary Figure S2: (A) Transcriptional starting sites of TAZ in MCF7 cells. ChIP-seq data were extracted from Cistrome database and presented to show TAZ gene. (**B**) TAZ promoter possessed a SRF CArG box. The position of CArG box in the TAZ promoter and the CArG box mutant were indicated.
